# Draft Genome Sequences of Pandrug-Resistant *Serratia marcescens* Clinical Isolates Harboring *bla_NDM-1_*

**DOI:** 10.1128/genomeA.01481-16

**Published:** 2017-01-19

**Authors:** Yancheng Yao, Linda Falgenhauer, Volkhard A. J. Kempf, Michael Hogardt, Stephan Göttig, Can Imirzalioglu, Trinad Chakraborty

**Affiliations:** a Institute of Medical Microbiology, German Center for Infection Research (DZIF Partner Site Giessen-Marburg-Langen), Justus-Liebig University Giessen, Giessen, Germany; b Institute for Medical Microbiology and Infection Control, University Hospital, Goethe-University, Frankfurt am Main, Germany

## Abstract

The draft genome sequences of two clonal, pandrug-resistant *Serratia marcescens* clinical isolates were determined. The resistance phenotype was plasmid driven, as 14 of 17 resistance genes were present on large IncFIB(K), IncHI2, and IncA/C2 plasmids indicating a large pool of transmissible antibiotic resistance genes.

## GENOME ANNOUNCEMENT

*Serratia marcescens* is an environmental organism and colonizer of the gastrointestinal tract of healthy individuals. However, now it is increasingly being recognized as an emerging pathogen, causing severe infections and outbreaks particularly affecting preterms and neonates (1–3).

*S. marcescens* displays intrinsic resistance to ampicillin and colistin, however, clinical isolates of *S. marcescens* with pandrug resistance are rare (3, 4). The pandrug-resistant *S. marcescens* isolates SM1978 and SM1890 harboring *bla*_*NDM-1*_ obtained from a leukemia patient and a patient with a urinary tract infection, respectively, were previously described (5). We performed whole-genome sequencing and searched for the location of antibiotic resistance genes in these isolates.

Genomic DNA sequencing libraries were prepared using the Nextera XT kit (Illumina, Eindhoven, NL). Sequencing was performed using Nextseq Mid-output reagent kit v2 (2 × 150 bp) on an Illumina NextSeq 500.

For SM1890, a total of 6,655,202 reads with an average length of 134 bp were assembled into 174 contigs, giving a total length of 5,865,102 bp and a coverage of roughly 118× (CLC Genomics Workbench 9.0). Plasmid replicons and antibiotic resistance genes were predicted using the CGE platform (https://cge.cbs.dtu.dk/services/) (6, 7). Chromosomal contigs were ordered to the best matched reference strain WW4 (8) using MAUVE (9) and annotated with RAST (http://Rast.nmpdr.org) (10). The gapped draft chromosome of SM1890 comprised 5,358,595 bp with a G+C content of 59.1% and 132 contigs. In addition, contigs had homology to an IncA/C2 plasmid pNDM-KN (JN157804), IncHI2 plasmid p34977-263.138 kb (CP012170), and R478 (BX664015) as well as IncFIB(K) plasmid pKPN3 (CP000648). Three small closed plasmids designated pSM1890_ColRNA1_ (4,938 bp), pSM1890_ColRNA1_ (3,223 bp), and pSM1890_untypable_ (2,697 bp), respectively. The average sequencing depth was 70 for the chromosome, 120 for the three large plasmids, and from 11,000 to 13,000 for the three small plasmids. Analysis revealed *bla*_*SHV-12*_, *bla*_*TEM-1B*_, *bla*_*CMY-6*_, *sul1*, *sul2*, *rmtC*, *aacA4*, *aac*(*6′*)*Ib-c*, *strA*, *strB*, *dfrA18*, *qnrA1*, *catA2*, *aac*(*6′*)*-Ic, tet* (41), and *ampC* genes in addition to the *bla*_*NDM-1*_ gene. The majority of the antibiotic resistance genes are located on the IncA/C2, IncHI2A and IncFIB(K) plasmids, with only three (*aac*[*6′*]*-Ic, tet*[41], and *ampC*) present on the chromosome. The plasmid pSM1890_IncA/C2_ harbored the transmissible resistance genes *bla*_*NDM-1*_, *bla*_*CMY-6*_, *sul1*, *rmtC*, *aacA4*, and *aac(6′)Ib-c* (5). The IncHI2A and IncFIB plasmids also carried the operons *terABCDW*, *copBCD*, *arsCBRH* conferring resistance toward the heavy metals tellurium, copper, and arsenic. A notable finding was the presence of a gene with homology to methyl-accepting chemotaxis protein (MCP) on the 4,938 bp-ColRNA1 plasmid. The same gene present on plasmid pCSA2 of *Cronobacter sakazakii* ATCC29544 was previously shown to be involved in adhesion, invasion, and the regulation of mobility and biofilm formation in that isolate (11).

For SM1978 a total of 2,036,182 reads with an average length of 130 bp and assembled to a gapped genome length of 5,846,245 bp with ~44× coverage. The genome sequences of both isolates exhibited 99.99% ANI (average nucleotide identity).

The exceptional antibiotic resistance profile of these isolates can be attributed to an accumulation of multiple plasmids harboring a wide range of resistance determinants. As these plasmids can be horizontally transferred, these isolates constitute a reservoir for the spread of multidrug-resistance.

### Accession number(s).

Raw data and the assembled sequences have been deposited in the European Nucleotide Archive (ENA) under the accession no. FNXV00000000 for SM1890 and FNXW00000000 for SM1978. The versions described in this paper are the first versions, FNXV01000000 and FNXW01000000.
